# Antioxidant activity of lidocaine, bupivacaine, and ropivacaine in aqueous and lipophilic environments: an experimental and computational study

**DOI:** 10.3389/fchem.2023.1208843

**Published:** 2023-06-20

**Authors:** H. Kavčič, U. Jug, J. Mavri, N. Umek

**Affiliations:** ^1^ Clinical Department for Anesthesiology and Surgical Intensive Therapy, University Medical Center Ljubljana, Ljubljana, Slovenia; ^2^ Department of Anesthesiology and Reanimatology, Faculty of Medicine, University of Ljubljana, Ljubljana, Slovenia; ^3^ Department of Analytical Chemistry, National Institute of Chemistry, Ljubljana, Slovenia; ^4^ Laboratory of Computational Biochemistry and Drug Design, National Institute of Chemistry, Ljubljana, Slovenia; ^5^ Institute of Anatomy, Faculty of Medicine, University of Ljubljana, Ljubljana, Slovenia

**Keywords:** local anesthetic, free radical scavengers, antioxidant assays, lidocaine, peroxyl radical, density functional theory, lipid membrane

## Abstract

**Introduction:** Local anesthetics are widely recognized pharmaceutical compounds with various clinical effects. Recent research indicates that they positively impact the antioxidant system and they may function as free radical scavengers. We hypothesize that their scavenging activity is influenced by the lipophilicity of the environment.

**Methods:** We assessed the free radical scavenging capacity of three local anesthetics (lidocaine, bupivacaine, and ropivacaine) using ABTS, DPPH, and FRAP antioxidant assays. We also employed quantum chemistry methods to find the most probable reaction mechanism. The experiments were conducted in an aqueous environment simulating extracellular fluid or cytosol, and in a lipophilic environment (*n*-octanol) simulating cellular membranes or myelin sheets.

**Results:** All local anesthetics demonstrated ABTS˙^+^ radical scavenging activity, with lidocaine being the most effective. Compared to Vitamin C, lidocaine exhibited a 200-fold higher half-maximal inhibitory concentration. The most thermodynamically favorable and only possible reaction mechanism involved hydrogen atom transfer between the free radical and the -C-H vicinal to the carbonyl group. We found that the antioxidant activity of all tested local anesthetics was negligible in lipophilic environments, which was further confirmed by quantum chemical calculations.

**Conclusion:** Local anesthetics exhibit modest free radical scavenging activity in aqueous environments, with lidocaine demonstrating the highest activity. However, their antioxidant activity in lipophilic environments, such as cellular membranes, myelin sheets, and adipose tissue, appears to be negligible. Our results thus show that free radical scavenging activity is influenced by the lipophilicity of the environment.

## 1 Introduction

Reactive oxygen species (ROS) are a class of highly reactive chemicals formed by partial oxygen reduction. These include free radicals such as the peroxyl radical (RO^·^) and the hydroxyl radical (HO^·^), as well as molecules that have a high tendency to produce free radicals, such as hydrogen peroxide (H_2_O_2_) and the superoxide anion (O^−^) (Gulcin, 2020). ROS are produced continuously in biological systems through mitochondrial metabolism or as a response to external stimuli. They are present in low concentrations in all healthy cells and play a role in defense mechanisms and signaling pathways (Gulcin, 2020). ROS concentrations are regulated by various antioxidant systems, directly scavenging the radicals or indirectly modulating their activity. If this balance is disrupted, such as by a malfunctioning antioxidant system or an increase in free radical production due to environmental stress, the relative burden of free radicals begins to damage cellular components (Phaniendra et al., 2015). This damage can manifest as oxidation of DNA, peroxidation of lipids, inactivation of proteins and enzymes, and the promotion of tumor growth and inflammation. This pathological process is known as oxidative stress and has been implicated in various degenerative diseases, including Parkinson’s disease, Alzheimer’s dementia, and certain types of cancer (Phaniendra et al., 2015; Pavlin et al., 2016; Korovesis et al., 2023). Furthermore, cumulative damage caused by ROS is thought to play a significant role in aging (Phaniendra et al., 2015) and is believed to contribute to diabetes-induced endothelial dysfunction and the development of vascular complications such as atherosclerosis (Maruhashi and Higashi, 2021).

Local anesthetics are commonly used in medical and dental practice for pain control during surgical procedures and postoperative treatment (Helander et al., 2019). They exert their effects by blocking neural signal transduction along the axon via inhibition of voltage-dependent sodium channels (Kaplan et al., 1997). In addition to their local anesthetic effects, lidocaine has also been shown to have antiarrhythmic properties. Emerging evidence suggests that local anesthetics may also exert various other biological effects, such as chronic pain control (Helander et al., 2019), anticancer properties (Zheng et al., 2018), antibacterial activity (Johnson et al., 2008), anti-inflammatory effects (Hollmann et al., 2000), and antioxidant activity (Jae et al., 2010). Some experimental studies have reported a positive impact of local anesthetics on various antioxidant systems (Hara et al., 1993; Jae et al., 2010). For instance, Jae et al. reported that local anesthetics reduce the effect of reactive oxygen species-induced endothelial dysfunction in the rabbit abdominal aorta *in vitro* at concentrations greater than 300 µM (Jae et al., 2010).

Moreover, Hara et al. demonstrated that lidocaine protects isolated rat hearts from changes caused by peroxyl radicals at concentrations of 50 or 200 µM (Hara et al., 1993). Studies comparing the antioxidant activity of different local anesthetics are relatively scarce. However, Lenfant et al. showed that lidocaine, bupivacaine, and ropivacaine all reduced ROS-induced hemolysis of human erythrocytes *in vitro*, with lidocaine being the most effective at the lowest concentration of 50 μg/mL (Lenfant et al., 2004). Conversely, when lidocaine was administered intraperitoneally, increased ROS production, glutathione antioxidant system dysfunction, and lipid peroxidation were observed in rat brain nerve cells (Cano-Europa et al., 2008). Results of studies investigating the free radical scavenging activity of local anesthetics are also inconclusive. Lidocaine has been shown to act as a scavenger of several radicals; however, the results are dependent on the laboratory method used. For example, one study demonstrated lidocaine’s ability to scavenge the superoxide anion (Gunaydin and Demiryurek, 2001), while another failed to replicate these results but did demonstrate its scavenging properties for hydroxyl radical and singlet oxygen (Das and Misra, 1992). A comparison of the antioxidant efficacy of lidocaine, bupivacaine, and ropivacaine with vitamin E using the allophycocyanin assay failed to demonstrate efficacy (Lenfant et al., 2004). The inconsistent results from these studies may be attributed to the different mechanisms of free radical scavenging reactions in aqueous or lipophilic media. Since local anesthetics are highly lipophilic and accumulate significantly in lipid-rich compartments such as biological membranes and myelin sheets, their antioxidant activity in a lipophilic environment might be biologically important.

Accordingly, this study aimed to investigate whether three local anesthetics - lidocaine, bupivacaine, and ropivacaine - can act as free radical scavengers both in aqueous and lipophilic environments, simulating extracellular fluid or cytosol and cellular membranes, respectively. In addition, the most probable reaction mechanisms were further explored using quantum chemical calculations.

## 2 Materials and methods

### 2.1 Antioxidant laboratory assays

#### 2.1.1 Chemicals and materials used

Methanol (HPLC grade) was purchased from Honeywell Reagents (Seelze, Germany). Acetic acid (100% glacial), hydrochloric acid (37%), and ammonia solution (32%) were purchased from Merck (Darmstadt, Germany). *n-*octanol (99.5%), ammonium acetate (98.1%), ascorbic acid (99.5%), ⍺-tocopherol formulation (T 4389; approximately 400 mg ⍺-tocopherol per Gram), 2,3,5-triphenyl tetrazolium chloride (TPTZ, 98%), iron (III) chloride hexahydrate (98%), potassium persulfate (99%), 2,2-diphenyl-1-picrylhydrazyl (DPPH; 95%), lidocaine (98%) and ropivacaine (100%) were obtained from Sigma-Aldrich (Steinheim, Germany). 2,2′-Azino-bis(3-ethylbenzothiazoline-6-sulfonic acid) diammonium salt (ABTS, 99%) was purchased from Biochemics, Fluka (Steinheim, Germany). Bupivacaine (99%) was purchased from Biosynth Crbosynth (Compton, United Kingdom). Lidocaine Chlorhydrate (400 mg/20 mL, LOT 18T1397) was obtained from Braun. Ropivacaine Hydrochloride (75 mg/10 mL, LOT 12PCA07) and Levobupivacaine hydrochloride (50 mg/10 mL, LOT 18T1397) were obtained from Fresenius Kabi. A Milli-Q water purification system (Millipore, Bedford, Massachusetts, United States) was used to obtain ultrapure water (18 MΩ cm^−1^). Disposable plastic cuvettes were purchased from Brand (Wertheim, Germany).

#### 2.1.2 Preparation of reagents

The ABTS˙^+^ radical was generated by combining ABTS with a strong oxidizing agent, potassium persulfate. The procedure was taken from the literature (Re et al., 1999) with certain modifications specified in Supplementary Material. The FRAP reagent was prepared according to the standard method (Benzie and Strain, 1999). The DPPH assay was performed according to the method of Sharma and Bhat 2009, with some modifications. The exact procedures for the preparation of reagents are described in the Supplementary Material.

#### 2.1.3 ABTS, FRAP, and DPPH assays

The detailed preparation of calibration solution mixtures, test mixtures, negative controls, and “spectrophotometer blanks” is presented in the (Supplementary Table S1). All mixtures were prepared in dark glass vials, mixed by vortexing (5s), and evaluated spectrophotometrically (Lambda 45 UV/Vis spectrometer, Perkin Elmer, Waltham, Massachusetts, United States) at 734 nm for ABTS assay (decrease in absorbance shows antioxidant activity–radical quenching), at 593 nm for FRAP assay (increase in absorbance shows antioxidant activity–reduction of Fe^3+^ to Fe^2+^), and at 517 nm for DPPH assay (decrease in absorbance shows antioxidant activity–radical quenching).

The spectrophotometric evaluation was performed after incubating mixtures in the dark at room temperature: in the case of ABTS assay after 30 min and after 2 h (ropivacaine and bupivacaine), while in the case of FRAP and DPPH assays after 30 min and 16 h.

Vitamin C (ascorbic acid) was used as a reference standard for assays in aqueous media. Calibration solutions were prepared with the following concentrations of vitamin C (µM) in water: 100, 50, 10, 1, 0.1, and 0.01. Tocopherol formulation was used as a reference standard in octanolic media. Calibration solutions were prepared with the following concentrations of tocopherol formulation (mg/mL) in *n*-octanol: 10, 1, 0.1, 0.01, 0.001, 0.0001, and 0.00001. All assays were performed with slight modifications of original procedures (Benzie and Strain, 1999; Re et al., 1999; Gupta et al., 2018) to increase the sample-to-reagent ratio.

Local anesthetics in powder form (pure local anesthetics) were dissolved in water at the highest possible concentrations according to their solubility: lidocaine, 0.35 mg/mL; ropivacaine, 0.20 mg/mL; bupivacaine, 0.10 mg/mL, and tested for antioxidant activity using all three assays. ABTS assays of pure lidocaine aqueous solutions at t = 30 min (time from the preparation of the solutions to their exposure to the reagent) were performed in the lidocaine concentration range of 0.007–0.35 mg/mL in two parallel experiments to determine the half-maximal inhibitory concentration (IC_50_). We also tested the stability of antioxidant activity by exposing the dissolved lidocaine to light and room temperature for 20 h. ABTS assay was then performed in the same sequence as before in the concentration range of 0.007–0.35 mg/mL in two parallel experiments. Pure local anesthetics were dissolved in *n*-octanol at a concentration of 10 mg/mL, and modified ABTS assays in octanolic medium were performed in the local anesthetic concentration range of 0.00001–10 mg/mL to determine the IC_50_ values of the antioxidant activity. If necessary, the ultrasound chamber was used to increase the solubility of the pure local anesthetics. The IC_50_ values were calculated, and the curves were plotted in GraphPad Prism 9 (GraphPad Prism, 2023). The ABTS˙^+^ scavenging effect for all tested samples was calculated with the following equation (Li et al., 2009);

$$ABTS^{•+}\;scavenging\;effect\;\left(\%\right)=100-\left(A_{A}\times 100/A_{C}\right)$$ (1)

where A_A_ stands for the absorbance of the test mixture and A_C_ stands for the absorbance of the negative control.

Commercial medical grade local anesthetics, used in clinical practice–aqueous solutions of local anesthetics in the form of salts, lidocaine hydrochloride (20 mg/mL), ropivacaine hydrochloride (7.5 mg/mL), and levobupivacaine hydrochloride (5 mg/mL) were tested by ABTS assay without manipulating the samples and after adjusting their pH with NaOH solution. Lidocaine hydrochloride with a concentration of 10 mg/mL at pH = 7.4 was diluted with water, and ABTS assay was performed in the lidocaine concentration range of 0.00001–10 mg/mL to determine the IC_50_ value of the antioxidant activity.

The Vitamin C equivalent antioxidant capacity (VCEAC) was determined as the amount of Vitamin C in mg that exhibits the same antioxidative activity as 100 g (10^5^ mg) of the tested compound. It was calculated from the ratio of IC_50_ values.

$${VCEAC}_{LA}=\frac{{IC50}_{Vitamin\;C}}{{IC50}_{LA}}\times 10^{5}$$ (2)

### 2.2 Computational evaluation of plausible reaction mechanisms

We studied chemical reactions between ROS and local anesthetics by quantum chemical methods to provide for a postulated mechanism reaction free energy.

Chemical reactions proceed in the direction of lower free energy, *i.e.,* products must have lower free energy than the reactants;

$$\Delta G=\Delta G_{products}-\Delta G_{reactants}<0$$ (3)

For the reaction to happen in a reasonable amount of time, it also requires a reasonably low activation free energy. Since most reactions involving radical species in biological systems happen almost instantly, we assumed they all have considerably low activation free energy (Hawkins and Davies, 2001). Therefore, throughout this work, we focused on reaction free energy change (ΔG) to find the thermodynamically favored mechanism of free radical scavenging activity.

To find ΔG, we employed quantum chemical methods, a methodology successfully used to determine the antioxidant potential of various compounds in previous studies (Galano and Alvarez-Idaboy, 2019; Alisi et al., 2020; Pérez-González et al., 2020). We examined the reactions between three commonly used local anesthetics (lidocaine, bupivacaine, and ropivacaine) and hydroperoxyl radical (HOO^·^), a free radical commonly produced in human cells. Hydroperoxyl radical is often used in quantum chemical calculations of the antioxidative activity of different compounds due to its selectivity and stability (Galano et al., 2012; Caicedo et al., 2014; Alisi et al., 2020). It is also one of the main initiators of lipid peroxidation (Phaniendra et al., 2015). The calculations were performed for the reactions in an aqueous solution and in *n*-octanol, an established model for cell membranes in biochemical chemistry, provided limitations are accounted for (Leo et al., 1971; Sanchez et al., 1987). Solvent effects were included on the level of the solvent reaction field.

Free radical scavengers, also known as antioxidants, are molecules that neutralize the unpaired electron configuration of free radicals by accepting or donating electrons. In doing so, they may become less active or less dangerous new free radicals compared to the neutralized ones. The mechanism of scavenging by antioxidants typically also involves intermolecular proton/hydrogen atom transfer. Three distinct mechanisms have been proposed to account for this process: hydrogen atom transfer (HAT), single electron transfer followed by proton transfer (SET-PT), and sequential proton loss electron transfer (SPLET) (Alisi et al., 2020).

HAT is a chemical reaction consisting of the concerted movement of two elementary particles forming a hydrogen atom in its radical form (proton and electron) between two reactants in a single kinetic step (Alisi et al., 2020). The reaction free energy for the HAT (ΔG_HAT_) mechanism is the difference between the free energy of products (hydrogen peroxide and local anesthetic radical) and reactants (local anesthetic and hydroperoxyl radical).

$$\Delta G_{HAT}=\left(G\left[LA˙\right]+G\left[HOOH\right]\right)-\left(G\left[LA\right]+G\left[HOO˙\right]\right)$$ (4)

**FIGURE 1:**
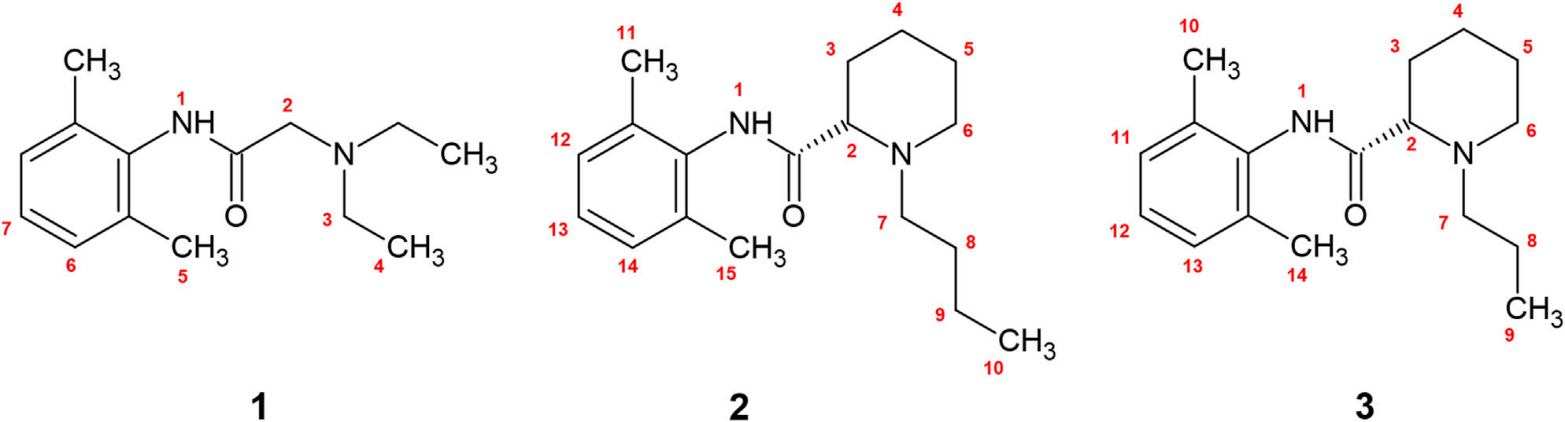
The numbered bonds of shown local anesthetics represent all scissile -N-H and -C-H bonds available for free radical scavenging. 1—lidocaine, 2—bupivacaine, 3—ropivacaine.

Please note that we considered all scissile -C-H and -N-H bonds in local anesthetics, as shown in Figure 1. At this point it is worth to emphasize that the HAT mechanism involves no ionic species and the charge distribution does not change significantly during the reaction. In this respect weak solvent effect on the reaction profile is anticipated. This can be contrasted with the electron and proton transfer reactions where kinetics is significantly solvent dependent.

The SET-PT mechanism involves a two-step reaction: in the first step, the antioxidant accepts the electron from the free radical, neutralizing it.

$$\Delta G_{SET-PT\;1}=\left(G\left[{LA˙}^{+}\right]+G\left[{HOO}^{-}\right]\right)-\left(G\left[LA\right]+G\left[HOO˙\right]\right)$$ (5)

In the next step, the antioxidant transfers a proton to the neutralized free radical, becoming a less reactive, stable radical in the process.

$$\Delta G_{SET-PT\;2}=\left(G\left[LA˙\right]+G\left[HOOH\right]\right)-\left(G\left[{LA˙}^{+}\right]+G\left[{HOO}^{-}\right]\right)$$ (6)

The SPLET mechanism also involves a two-step reaction: in the first step, the local anesthetic donates a proton to the neutralized anion radical.

$$\Delta G_{SPLET\;1}=\left(G\left[{LA}^{-}\right]+G\left[HOOH\right]\right)-\left(G\left[LA\right]+G\left[{HOO}^{-}\right]\right)$$ (7)

In the second step, the anion antioxidant donates the electron to the free radical, neutralizing it.

$$\Delta G_{SPLET}=\left(G\left[LA˙\right]+G\left[{HOO}^{-}\right]\right)-\left(G\left[{LA}^{-}\right]+G\left[HOO˙\right]\right)$$ (8)

**Abbreviations in equations**: ΔG [LA^·^], Gibbs free energy of local anesthetic radical; ΔG [HOOH], Gibbs free energy of peroxide; ΔG [LA], Gibbs free energy of neutral local anesthetic; ΔG [HOO^·^], Gibbs free energy of hydroperoxyl radical; ΔG [LA^·+^], Gibbs free energy of local anesthetic radical cation; ΔG [HOO^−^], Gibbs free energy of free radical anion; ΔG [LA^−^], Gibbs free energy of local anesthetic anion.

Since experimental evidence which hydrogen atom in the local anesthetic molecule is involved in the reaction is limited, we performed calculations for all of them. The enumeration for all possible scissile -C-H and -N-H bonds along with the structures are shown in Figure 1. The structural schemes of the reactions can be found in the (Supplementary Figures S1–S3).

#### 2.2.1 Quantum chemical calculations

We performed quantum chemical calculations of the reactions between local anesthetics and the peroxyl radical. The structures of all local anesthetics, free radicals, and the products of the reactions were built using the Molden v5.8 software package (Schaftenaar and NoordikMolden, 2000). The calculations were performed using the Gaussian 16 software package (Frisch et al., 2016) using the Minnesota M06-2X functional in the framework of Density Functional Theory (DFT) (Zhao and Truhlar, 2008). The theory of electron and proton transfer processes is established and available for application (Olsson et al., 2006). To ensure the reliability of our results, the initial geometries of the structures were optimized at the M06-2X/6-311+G (d,p) level, which is a balance between computational cost and accuracy. This method includes the long-range effects of unpaired electrons and has been widely used in studies of free radical reactions in quantum chemistry (Pandithavidana and Jayawardana, 2019; Alisi et al., 2020). The effects of solvation were considered by applying the universal solvation model (SMD) (Marenich et al., 2009). We chose the SMD solvation model because, in our previous studies, it was found to be the most accurate in reproducing the solvation effects of local anesthetics and other organic molecules (Umek, 2020; Kavčič et al., 2021a; Umek, 2022). In this study, we considered two solvents: water with a dielectric constant of 78.30 and n-octanol with a dielectric constant of 9.86. Inclusion of the solvent reaction field to the quantum-chemical calculations gives the Born-Oppenheimer surface meaning as a free energy surface. A vibrational analysis was performed in the harmonic approximation for all minimized structures. The calculated frequencies allowed for thermodynamic corrections of free energies at 298.15 K.

## 3 Results

### 3.1 Antioxidant activity determined with standard antioxidant activity assays

#### 3.1.1 Antioxidant activity of pure local anesthetics

Lidocaine (0.35 mg/mL) showed antioxidant activity 30 min after exposure to the ABTS reagent. In contrast, ropivacaine (0.20 mg/mL) and bupivacaine (0.10 mg/mL) showed only a slight antioxidant activity 30 min after exposure to the reagent (Table 1). The assay was also performed 2 h after exposure of bupivacaine and ropivacaine to the ABTS reagent (Table 1), where they caused almost complete decolorization of the ABTS reagent, which was confirmed spectrophotometrically. We did not repeat the test after 2 h with lidocaine because the sample had been discolorized already after 30 min.

**TABLE 1:** ABTS assay of antioxidant activity of pure local anesthetics in an aqueous medium.

Local anesthetic	Concentration mg/mL	Absorbance values		ABTS˙^+^ scavenging effect in %	
		After 30 min	After 2 h	After 30 min	After 2 h
Lidocaine	0.35	0.0054	N/P	97.81	N/P
Bupivacaine	0.10	0.1010	0.0479	59.06	81.71
Ropivacaine	0.20	0.0854	0.0334	65.38	87.25
Negative control	0	0.2467	0.2619	N/A	N/A

ABTS˙^+^ scavenging effect was calculated using Eq. 1. N/A–not applicable. N/P–not performed.

We tested the antioxidant potential of all three antioxidants by DPPH and FRAP assays, using vitamin C as a reference standard. The results did not show any antioxidant activity and are summarized in (Supplementary Tables S2, S3).

Because lidocaine proved to be the most promising antioxidant among the tested local anesthetics, the half-maximal inhibitory concentration (IC_50_) of the antioxidant activity of pure lidocaine was determined (Table 2), and the stability of antioxidant activity after 20 h was confirmed. The determined antioxidant potential was about 60-fold lower compared with the vitamin C reference standard according to the IC_50_ values.

**TABLE 2:** Half maximal inhibitory concentration of lidocaine compared to reference standard in an aqueous medium using the ABTS assay.

Medium	Local anesthetics and References standards	IC_50_ [mg/mL]	Log IC_50_	Hillslope	VCEAC [mg VitC/100 g]
Aqueous	Pure dissolved lidocaine	0.09092	−1.041	1.757	1,635
	Lidocaine hydrochloride, buffered to pH 7.4	0.3311	−0.4801	1.002	449
	Vitamin C (references)	0.001487	−2.828	1.508	N/A

IC_50_ - half maximal inhibitory concentration, VCEAC-Vitamin C mg/100 g equivalent antioxidant capacity (calculated using Eq. 2). N/A-not applicable.

**FIGURE 2:**
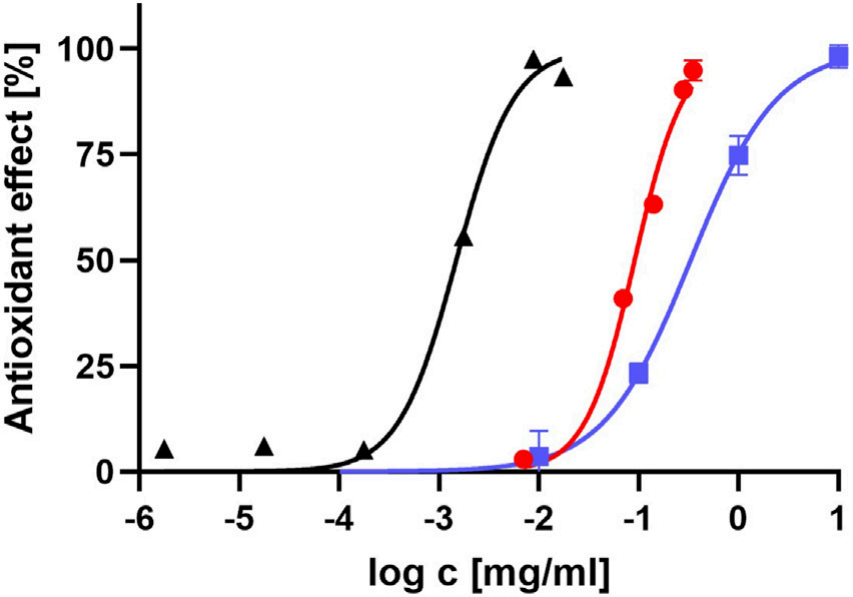
Antioxidant effect of lidocaine, compared to vitamin C. The graph represents how antioxidant effect of pure dissolved lidocaine (red dots), lidocaine hydrochloride, buffered to pH value of 7.4 (blue squares) and vitamin C (black triangles) is related to the logarithm of their concentration.

We compared the log IC_50_ results between the pure dissolved lidocaine and lidocaine hydrochloride, buffered to a pH of 7.4, to the reference standard of vitamin C. The results are displayed in Figure 2.

The Vitamin C mg/100 g equivalent (VCEAC) for pure lidocaine and lidocaine hydrochloride, buffered to a pH of 7.4, was calculated from Eq. 2. The results are summarized in Table 2.

To determine if the antioxidant activity of local anesthetics is retained in lipophilic compartments such as cellular membranes, we tested several local anesthetics by the modified ABTS assay in *n-*octanol solution. All tested local anesthetics showed very low antioxidant activity that was about 1,000 times lower compared to the tocopherol reference standard (Table 3).

**TABLE 3:** Half maximal inhibitory concentration of local anesthetics compared to reference standard in octanolic medium using the ABTS assay.

Medium	Local anesthetics and References standards	IC_50_ [mg/mL]	log IC_50_	Hillslope
*n-*octanol	Lidocaine	1.664	0.2212	2.168
	Ropivacaine	1.908	0.2806	1.549
	Bupivacaine	1.860	0.2695	1.722
	Tocopherol (reference)	0.006713	−2.173	1.207

IC_50_ - half maximal inhibitory concentration.

#### 3.1.2 Antioxidant activity of commercial formulations of local anesthetics

To determine the antioxidant activity of local anesthetic preparations used in clinical practice, we tested samples of commercial formulations of 2% lidocaine hydrochloride, 0.5% bupivacaine hydrochloride, and 0.75% ropivacaine hydrochloride. Since FRAP and DPPH tests did not show any antioxidant activity of local anesthetic solutions, we repeated the test only with the ABTS assay. Although the solutions were tested at high concentrations, they showed low antioxidant activity (Table 4), even lower than aqueous solutions of pure local anesthetics. Again, lidocaine hydrochloride showed the highest antioxidant potential.

**TABLE 4:** ABTS assay of antioxidant activity of commercial formulations of local anesthetics in an aqueous medium.

Local anesthetic	Concentration (mg/mL)	Absorbance values		ABTS˙^+^ scavenging effect (%)	
		After 30 min	After 2 h	After 30 min	After 2 h
Lidocaine hydrochloride	20	0.0249	0.0172	90.13	93.04
Bupivacaine hydrochloride	5	0.1383	0.1048	45.16	57.62
Ropivacaine hydrochloride	7.5	0.1324	0.1048	47.50	57.62
Lidocaine hydrochloride buffered to 7.4 pH	10	0.00395	N/P	96.20	N/P
Negative control	0	0.2522	0.2473	N/A	N/A

ABTS˙^+^ scavenging effect was calculated using Eq. 1. N/A–not applicable. N/P–not performed.

To simulate the biological environment where local anesthetics exert their antioxidant activity, we tested the commercial formulation of local anesthetics at the physiological pH value of 7.4. The pH values of the anesthetic solutions were measured (lidocaine hydrochloride, 6.06; ropivacaine hydrochloride, 5.59; bupivacaine hydrochloride, 5.65), and NaOH solution was added to obtain a physiological pH of 7.4. Bupivacaine hydrochloride and ropivacaine hydrochloride precipitated, while the lidocaine hydrochloride solution was clear at pH 7.4 (Table 4). While adjusting the pH, we diluted the original lidocaine hydrochloride solution to a concentration of 10 mg/mL and performed an ABTS assay in the range of 0.00001–10 mg/mL. We obtained absorbance of IC_50_ of 0.3311 and log IC_50_ of −0.4801, with IC_50_ value slightly higher compared to the pure lidocaine (Table 2).

### 3.2 Reaction mechanisms of antioxidant activity

Using quantum chemical calculations, we showed that in an aqueous solution, the reaction between the radical and the -C-H bond at position 2 (Figure 1) was the only one resulting in negative reaction free energy, indicating a thermodynamically favorable reaction (Table 5), while in *n-*octanol no reaction between local anesthetic and peroxyl radical was thermodynamically favorable at any position (Table 5). The complete results can be found in the (Supplementary Tables S4–S6).

**TABLE 5:** Reaction free energy of the reaction between local anesthetics and peroxyl radical on position 2 in water and *n-*octanol.

Local anesthetic [in water]	Mechanism				
	HAT	SEP-PT		SPLET	
	ΔG_HAT_	ΔG_SEP-PT 1_	ΔG_SEP-PT 2_	ΔG_SPLET 1_	ΔG_SPLET 2_
Lidocaine	−3.16	22.91	−26.06	20.4	−23.55
Bupivacaine	−0.24	37.86	−38.1	29.72	−29.96
Ropivacaine	−1.71	37.4	−39.11	28.55	−30.26
**Local anesthetic [in *n-*octanol]**					
Lidocaine	16.44	40.1	−23.66	28.68	−12.24
Bupivacaine	3.32	41.96	−38.63	10.76	−7.44
Ropivacaine	3.5	41.93	−38.43	10.61	−7.11

The values for the free energy of the reaction were calculated using the Minnesota functional of DFT, at the level of theory M06-2X/6–311+G(d,p) and SMD, solvation model. All results are calculated in kcal/mol and represent the reaction free energy. HAT, hydrogen atom transfer; SEP-PT, single electron transfer followed by proton transfer; SPLET, sequential proton loss electron transfer. Position 2 is the -C-H bond next to the carbonyl group (Figure 1).

The most plausible reaction mechanism was HAT. Both SEP-PT and SPLET mechanisms are also thermodynamically possible in water because the overall difference of both steps is equally negative. However, the large endothermicity of Step 1 of the reaction for all local anesthetics in water represents a very high energy barrier, which means that this reaction is extremely slow and not clinically relevant. Please note that the corresponding barriers in *n-*octanol are even higher than in water.

## 4 Discussion

In the present study, we showed that local anesthetics exhibit a modest radical scavenging antioxidant activity in an aqueous environment, which was further supported by a thermodynamically favorable reaction between the free radical and the -C-H group on position 2 (Figure 1) with hydrogen atom transfer being the most plausible reaction mechanism. Among studied local anesthetics, lidocaine exhibited the highest antioxidant activity. Furthermore, we demonstrated that the antioxidant activity of the tested local anesthetics was negligible in a lipophilic environment, which was also confirmed by quantum chemical calculations.

### 4.1 Scavenging ability of lidocaine

Pure lidocaine in an aqueous solution displayed scavenging activity for the ABTS^·+^ radical. Stability of antioxidant activity was also confirmed. The commercial and clinically used hydrochloride salt form of lidocaine with pH value of 6.06 also exhibited antioxidant activity, though to a lesser extent. Local anesthetics are weak bases, changing their protonation status in accordance with the pH value of the environment. The neutral form of a weak base has more electrons available for donation and is, therefore, a better radical scavenger (Lemańska et al., 2001; Muzolf et al., 2008; Xie et al., 2018). The higher pH value of dissolved pure lidocaine could therefore contribute to its higher scavenging ability of the ABTS˙^+^ radical. Additionally, quantum chemical analysis showed that the most probable mechanism was hydrogen atom transfer between the -C-H group on position 2 of lidocaine and the peroxyl radical, resulting in the formation of a lidocaine radical and peroxide. This was the only thermodynamically plausible reaction path. Simulating reactions with ABTS˙^+^ radical in quantum chemistry calculations would take a huge amount of processing power, which is why we have not been able to correlate the experiments directly. Since reactions with ABTS˙^+^ radical preferably take the form of hydrogen atom transfer (Apak et al., 2016), similar to our calculated reaction with peroxyl radical, we believe that our study still provides important data regarding the local anesthetic antioxidative activity.

Molecules containing an aromatic ring, such as local anesthetics, are more polarizable, allowing for electrons to be delocalized over the entire ring instead of on a single atom. Therefore, they can succesfully scavange radicals, despite lacking a phenolic -O-H or amino bond, similar to carotenoids (Charlton et al., 2023). While antioxidative activity is usually expected at -O-H or -N-H bonds, it was found that -C-H bonds play a fundamental role in the antioxidant properties of specific flavonoids, hydrogen atom transfer being the most plausible mechanism (Vo et al., 2019). Considering these findings, we can propose that the observed antioxidant effect of lidocaine is at least partially attributed to its direct scavenging mechanism.

### 4.2 Effect of pH

The human body maintains the pH of plasma and extracellular fluid within a narrow range of 7.35–7.45 (Kellum, 2000). To simulate the physiological environment and reconcile differences between samples of different local anesthetics in their commercial formulations with different pH values, the hydrochloride salt solution of local anesthetics was buffered to a pH of 7.4. Increasing the pH of the commercial lidocaine solution improved its antioxidative activity (Table 4), which could partially be attributed to the increased scavenging ability of ABTS˙^+^ radical at higher pH values, as discussed before. The IC_50_ value of buffered lidocaine at physiological pH value was higher than that of the aqueous solution of pure powder yet still within the same range (Figure 2). Commercial solutions of local anesthetics contain a few adjuncts that could influence the reaction with the ABTS˙^+^ radical, contributing to the difference in results compared to the pure dissolved anesthetic. Since the disparity between both IC_50_ values is small (in the same range), it could also be attributed to error. Nevertheless, our results suggest that clinically used lidocaine solutions exhibit scavenging ability when buffered to the pH of extracellular fluid. Attempts to buffer commercial forms of bupivacaine and ropivacaine to pH 7.4 resulted in the precipitation of both compounds.

### 4.3 Scavenging ability of bupivacaine and ropivacaine

Both bupivacaine and ropivacaine demonstrated scavenging effects on ABTS assay, whether prepared as an aqueous solution of pure powder or as a clinically used formulation of the hydrochloride salt. However, their antioxidant activity was found to be weaker and slower in comparison to lidocaine. This suggests that the kinetics of the antioxidant reaction (radical scavenging reaction) for bupivacaine and ropivacaine are slower than that of lidocaine. It should also be noted that the reaction between vitamin C and the ABTS reagent was basically instantaneous in comparison. Additionally, slight variations in concentration were observed among the local anesthetics. These results are consistent with similar findings reported in the literature (Lenfant et al., 2004). Quantum chemical calculations indicated a plausible reaction between the -C-H group at position 2 (Figure 1) of bupivacaine or ropivacaine and peroxyl radical. Therefore, it is suggested that the most likely reaction mechanism with peroxyl radical in water is the HAT mechanism at position 2, similar to certain flavonoids (Apak et al., 2013) The pKa values of bupivacaine and ropivacaine are 8.21 and 8.16, respectively (Kavčič et al., 2021b). Compared to lidocaine, there is a higher proportion of protonated form of the molecule present at the same pH value in the environment. The protonated form is less likely to exhibit antioxidative activity due to decreased ease of electron donation (Lemańska et al., 2001).

### 4.4 Effect of lipophilic environment

The lipophilic compartment model suggests that the intramembrane concentration of lidocaine could be 42 times higher than the extracellular concentration due to its high degree of lipid solubility in its neutral form (Kavčič et al., 2021b). Please note that the lipid solubility of bupivacaine and ropivacaine is even higher. Lipid peroxidation, an important component of free radical cell damage, has been linked to the pathogenesis of several human diseases, including diabetic neuropathy (Gianazza et al., 2019). High concentrations of lidocaine in the membrane could inhibit the peroxidation chain reaction, providing a basis for its antioxidant effect. To further investigate the free radical reaction in a lipophilic environment, the membrane bilayer was simulated by *n-*octanol solution, an established approach in drug design. The ABTS assay and quantum chemical reaction field were adapted accordingly. As vitamin C is not soluble in *n*-octanol, a new standard of lipid-soluble vitamin E (tocopherol) was used, which exhibited the expected concentration/antioxidant activity relationship (Figure 2). Our results show that the antioxidant activity of all three local anesthetics is very low in the lipophilic environment. Compared to the tocopherol standard, they exhibited approximately 1,000 times lower activity. Quantum chemistry calculations in *n*-octanol also did not find any thermodynamically favorable reaction mechanism for all three tested local anesthetics, which is consistent with the very low antioxidant activity observed in the ABTS assay. We cannot provide a sound structural explanation for the negative results.

### 4.5 Relevance to clinical practice

Lidocaine demonstrated approximately 60-fold lower antioxidant activity compared to vitamin C (Figure 2). The VCEAC values of different forms of lidocaine are in the same range as Vitamin A and some weaker antioxidants, such as certain flavones (7-hydroxyflavone and rhoifolin), and various other compounds (hydroxybenzoic acid, salicylic acid) (Kim and Lee, 2004). Previous studies have also reported that lidocaine protected human erythrocytes from oxidative challenge at concentrations of 0.05 mg/mL, consistent with the results and concentrations used in the current study (Lenfant et al., 2004). All three local anesthetics are available in similar preparations commercially, with concentrations ranging from 0.125% to 0.5% for bupivacaine and 1%–2% for lidocaine (Helander et al., 2019). Neurological side effects have been reported for bupivacaine at venous concentrations of 2–3 μg/mL, and cardiovascular side effects become more likely at higher concentrations. For lidocaine, plasma concentrations above 6 or 10 μg/mL have been reported to cause adverse effects (Collinsworth et al., 1974). However, the concentrations of lidocaine needed to provide the same level of free radical scavenging as vitamin C would likely exceed the toxic concentration in plasma. While lidocaine could reach the necessary concentration in the membrane due to partitioning, its scavenging ability seems to diminish significantly in a lipophilic environment. Nevertheless, our study suggests lidocaine has a higher antioxidant activity than bupivacaine and ropivacaine, which was confirmed using two different methods. Consistent with our results, lidocaine was also more effective than bupivacaine and ropivacaine in protecting human erythrocytes from an oxidative challenge (Lenfant et al., 2004). Using a phycoerythrin fluorescence-based assay, lidocaine was similarly found to have greater antioxidant activity than bupivacaine (Kang et al., 1998). Many patients requiring therapy with local anesthetics for surgical reasons may have increased oxidative stress due to the procedure or comorbidities such as *diabetes mellitus* or heavy smoking. In these cases, using a local anesthetic with a higher antioxidant activity, such as lidocaine, may be beneficial.

### 4.6 DPPH and FRAP assays

We also performed the DPPH and FRAP laboratory assays for all three local anesthetics, which showed no antioxidant activity. FRAP test measures the ability of the tested antioxidant to reduce ferric iron, which is an electron transfer reaction (Apak et al., 2013). On the other hand, ABTS˙^+^ and DPPH radicals may be deactivated either by radical quenching via HAT or by direct reduction through electron transfer mechanisms (Apak et al., 2016; Dimić et al., 2017; Dimić et al., 2018). Our computational results suggest that HAT is the most plausible mechanism of the direct antioxidant activity of tested local anesthetics; therefore, FRAP assay might not be able to detect such activity. The DPPH assay is conducted under a methanol/water mixture, whilst the ABTS assay was carried out separately in aqueous media and a methanol/n-octanol mixture in our study. Results from ABTS assay in an n-octanol mixture show no antioxidative activity, which was corroborated by positive reaction-free energies predicted by quantum chemical calculations under similar conditions. We assume that the antioxidative activity of local anesthetics was diminished in a lipophilic environment. Since the reaction with DPPH is conducted in a lipophilic environment, this could be the reason behind the negative results in DPPH assay. Research shows that the ABTS assay usually estimates higher antioxidative activity than DPPH assay when compared under the same circumstances, with some molecules showing no DPPH radical quenching at all (Martysiak-Żurowska and Wenta, 2012; Lee et al., 2015). Antioxidative activity, determined by DPPH assay usually shows dependance on the number of hydroxyl groups (-O-H), whereas ABTS assay does not show the same structural-activity relationship (Dimić et al., 2017; Dimić et al., 2019; Platzer et al., 2021).

### 4.7 Limitations of the study

Our study has a few limitations. Antioxidant assays provide rapid results, but it should be noted that the antioxidant reaction observed *in vitro* may not accurately reflect the situation *in vivo*. The pharmacology of antioxidants should be considered, as the concentrations tested in antioxidant assays are usually higher than what would be present *in vivo*. Additionally, the radicals that react *in vivo* are not directly comparable to the stable high molecular mass free radicals used in antioxidant assays. It is also possible that the body’s chemistry may influence the antioxidant activity of local anesthetics, which would not be evident *in vitro* tests. Unclear chemistry and lack of standardization are other limitations of these assays (Schaich et al., 2015). This study focuses only on three local anesthetics, while several other compounds within the same chemical class exist. Furthermore, translating these findings to human disease research is also a limitation since the calculated IC_50_ value is close to the concentrations of local anesthetics, which have been reported to produce side effects in certain cases.

## 5 Conclusion

Our study demonstrates that in aqueous solution, lidocaine, bupivacaine, and ropivacaine demonstrate scavenging activity against the ABTS^·+^ radical, with lidocaine being the most potent. Antioxidant activities of the studied local anesthetics are modest; the determined antioxidant potential of lidocaine was about 60-fold lower compared with the vitamin C reference standard according to the IC_50_ values. These results agree with the quantum chemical calculations. The only plausible mechanism found was hydrogen atom transfer between the -C-H group, vicinal to the carbonyl group (Position 2 in Figure 1) of local anesthetics and peroxyl radical. However, in a lipophilic environment where lipophilic local anesthetics would have the highest concentrations *in situ*, no conclusive antioxidant activity was observed using either the ABTS assay method or quantum chemical calculations, which suggests that the antioxidant activity of local anesthetics is substantially lower in a lipophilic environment. These results highlight the importance of a multidisciplinary approach when studying free radical mechanisms. Further computational, experimental, and especially clinical studies are needed to better understand the role of direct and indirect antioxidant activity of local anesthetics *in vivo* and its importance for pathological states such as diabetes where ROS production is increased.

## Data Availability

The original contributions presented in the study are included in the article/Supplementary Material, further inquiries can be directed to the corresponding author.

## Abbreviations

A_A_, Absorbance of test mixture; ABTS assay, 2,2-Azino-bis(3-ethylbenzothiazoline-6-sulfonic acid) assay; A_C_, Absorbance of control; DPPH assay, 2,2-diphenyl-1-picrylhydrazyl assay; DFT, Density functional theory; FRAP assay, Ferric Reducing Antioxidant Power Assay; HAT, Hydrogen atom transfer; IC_50_, Half-maximal inhibitory concentration; LA, Local anesthetic; SET-PT, Single electron transfer followed by proton transfer; SMD, Universal solvation model, based on density; SPLET, Sequential proton loss electron transfer; VC, Vitamin C; VCEAC, Vitamin C mg/100 g equivalent antioxidative capacity; ΔG, Difference of Gibbs free energy.
