# Supplementary material for: Antioxidant activity of lidocaine, bupivacaine, and ropivacaine in aqueous and lipophilic environments: an experimental and computational study
Source: Front Chem. 2023 Jun 20;11:1208843. doi: 10.3389/fchem.2023.1208843 (PMC10318152; doi:10.3389/fchem.2023.1208843)
Supplement: Supplementary file 1 [file DataSheet1.docx]

Supplementary Material

Antioxidant activity of lidocaine, bupivacaine, and ropivacaine in aqueous and lipophilic environments: an experimental and computational study

**Hana Kavčič, Urška Jug, Janez Mavri, Nejc Umek***

*** Correspondence:** Nejc Umek: [nejc.umek@mf.uni-lj.si](mailto:nejc.umek@mf.uni-lj.si)

# Preparation of reagents

## ABTS

An aqueous solution of 2,2′-azino-bis (3-ethylbenzothiazoline-6-sulfonic acid) in the form of a diammonium salt (ABTS, 7 mM) was combined with an aqueous solution of potassium persulfate (2.45 mM) in a 1:1 (*v/v*) ratio, and after 16 h in the dark at room temperature, the solution was diluted 64-fold with water for assays in aqueous media or 8-fold with MeOH for assays in octanolic media (Re et al. 1999). The prepared reagents were stored in the dark at 5 °C.

## FRAP reagent

The FRAP reagent, freshly prepared 2 h prior to the FRAP assay, consisted of a 300 mM ammonium acetate buffer (pH 3.6), a 10 mM solution of 2,4,6-tripyridyl-S-triazine (TPTZ) in 40 mM hydrochloric acid, and 20 mM ferric (III) chloride in water, combined in a 10:1:1 (*v/v/v*) ratio. The acetate buffer was stored at room temperature, while ferric (III) chloride (in a plastic container) and TPTZ solutions were stored in the dark at 5 °C (Benzie and Strain 1999).

## DPPH

The prepared 200 µM methanolic solution of 2,2-diphenyl-1-picrylhydrazyl (DPPH) (Sharma and Bhat 2009) was stored in the dark at 5 °C.

# Preparation of ABTS, FRAP and DPPH assays

The detailed preparation of calibration solution mixtures, test mixtures, negative controls, and “spectrophotometer blanks” is presented in **Table 1S**:

**Table 1S. Compositions of mixtures used in ABTS, FRAP, and DPPH Assays**

|  | **ABTS** | | **FRAP** | **DPPH** |
| --- | --- | --- | --- | --- |
|  | **Aqueous media** | **Octanolic media** | **Aqueous media** | **Aqueous media** |
| **Calibration solution mixture**  (reagent + reference standard calibration solution) | 2 mL + 2 mL | 200 µL + 1400 µL | 1 mL + 3 mL | 200 µL + 3 mL |
| **Test mixture** (A_A_)  (reagent + sample solution) | 2 mL + 2 mL | 200 µL + 1400 µL | 1 mL + 3 mL | 200 µL + 3 mL |
| **Negative control**  (A_C_)  (reagent + sample solvent) | 2 mL + 2 mL | 200 µL + 1400 µL | 1 mL + 3 mL | 200 µL + 3 mL |
| **“Spectrophotometer blank”**  (reagent solvent +  sample solvent) | 2 mL + 2 mL | 200 µL + 1400 µL | 1 mL + 3 mL | 200 µL + 3 mL |

Compositions of calibration solution mixtures, test mixtures, negative controls, and “spectrophotometer blanks” used in ABTS, FRAP, and DPPH assays are listed. All solutions were prepared in water for assays performed in aqueous media, except the DPPH reagent prepared in methanol. For ABTS assays performed in octanolic media, all solutions were prepared in *n*-octanol, while the ABTS reagent medium was water: methanol = 1: 7 (*v/v*).

# Schemes of reactions

Three distinct mechanisms have been proposed to account for the scavenging process: hydrogen atom transfer (HAT), single electron transfer followed by proton transfer (SET-PT), and sequential proton loss electron transfer (SPLET). Figures 1S through 3S are visual representations of possible reaction paths between lidocaine and hydroperoxyl molecule.

Hydrogen atom transfer is a chemical reaction consisting of the concerted movement of a hydrogen atom between two reactants in a single kinetic step. It is represented in Figure 1S.

**Figure 1S:** Hydrogen atom transfer reaction


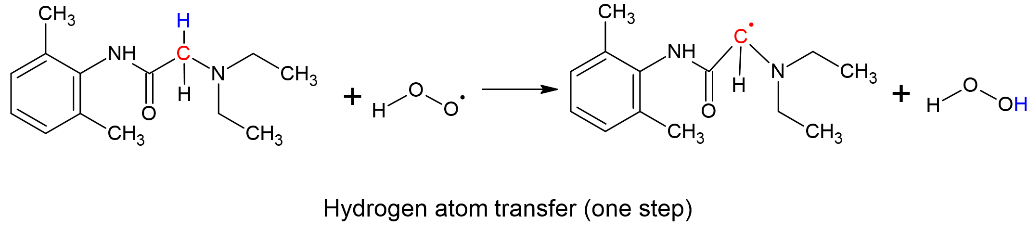


**Figure 1S caption:** Visual representation of hydrogen atom transfer from hydrogen atom on position 2 (refer to Figure 1) of lidocaine to the hydroperoxyl radical. The position 2 carbon atom is colored red.

The SET-PT mechanism involves a two-step reaction: in the first step, the antioxidant accepts the electron from the free radical, in the next step, the antioxidant transfers a proton to the neutralized free radical (Figure 2S).

**Figure 2S:** Single electron transfer followed by proton transfer reaction


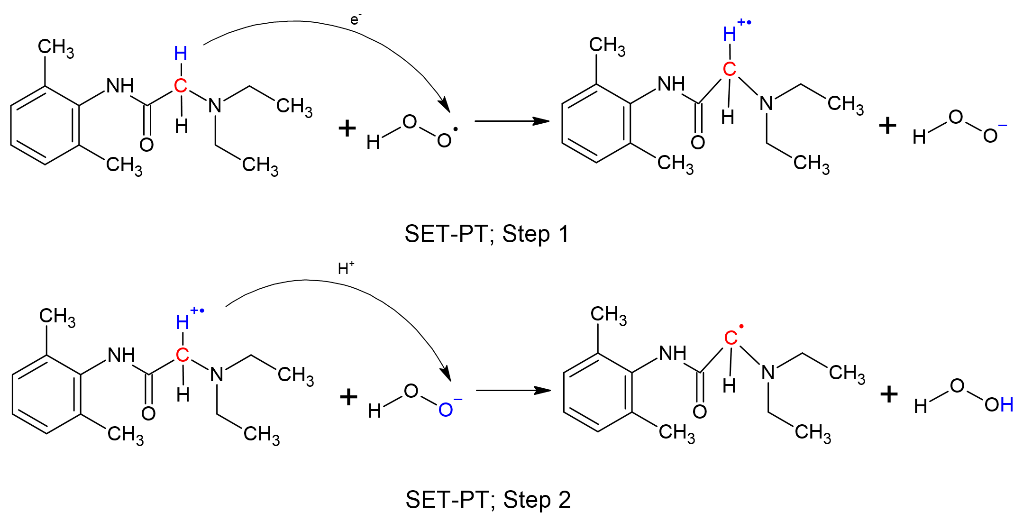


**Figure 2S caption**: Visual representation of single electron transfer followed by proton transfer between lidocaine and hydroperoxyl radical. The position 2 carbon atom is colored red.

The SPLET mechanism involves a two-step reaction: in the first step, the antioxidant donates a proton to the neutralized anion radical, in the second step, the anion antioxidant donates the electron to the free radical (Figure 3S).

**Figure 3S:** Sequential proton loss electron transfer reaction


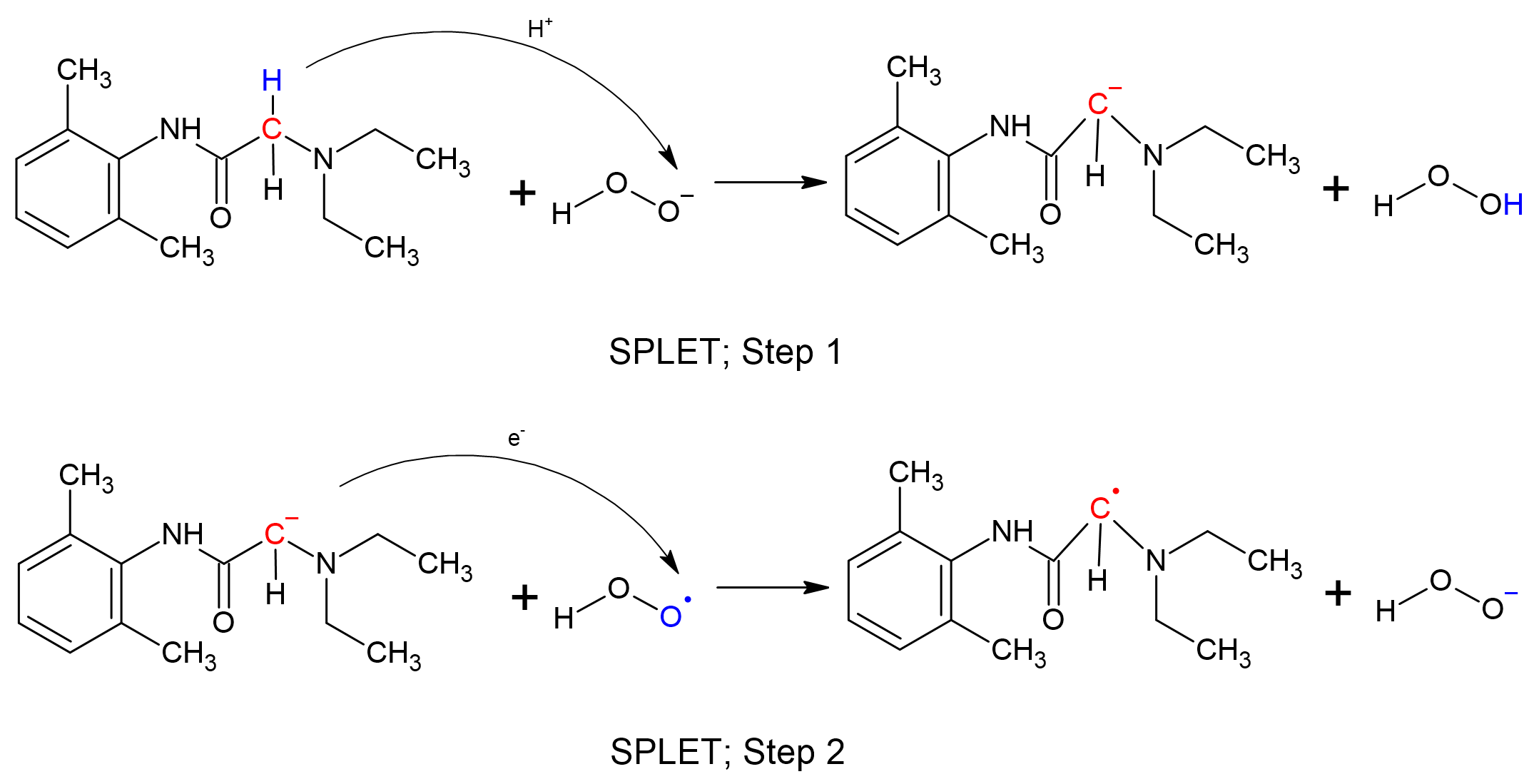


**Figure 3S caption**: Visual representation of sequential proton loss electron transfer between lidocaine and hydroperoxyl radical. The position 2 carbon atom is colored red.

# Results from DPPH and FRAP Assay

We performed the DPPH Assay on a sample of dissolved pure lidocaine, bupivacaine, and ropivacaine. The results are summarized in **Table 2S**. For the DPPH˙ radical, decrease in absorbance shows antioxidant activity. Our measurements showed no significant decrease in absorbance compared to the negative control after 30 minutes or 16 hours.

**Table 2S: DPPH assay of antioxidant activity of pure local anesthetics in aqueous medium**

|  |  | Absorbance values | |
| --- | --- | --- | --- |
| Local anesthetic | Concentration  mg/mL | After 30 min | After 16 h |
| Lidocaine | 0.35 | 0.1216 | 0.0828 |
| Bupivacaine | 0.10 | 0.1183 | 0.0874 |
| Ropivacaine | 0.20 | 0.1112 | 0.0815 |
| Negative control | 0 | 0.0943 | 0.1042 |
|  |  |  |  |

We also performed the FRAP Assay on a sample of dissolved pure lidocaine, bupivacaine, and ropivacaine. The results are summarized in **Table 3S**. FRAP test shows the antioxidant activity as an increase in absorbance, visibly turning from yellow to blue. Our measurements showed no significant increase in absorbance compared to the negative control after 30 minutes or 16 hours.

**Table 3S: FRAP assay of antioxidant activity of pure local anesthetics in aqueous medium**

|  |  | Absorbance values | |
| --- | --- | --- | --- |
| Local anesthetic | Concentration  mg/mL | After 30 min | After 16 h |
| Lidocaine | 0.35 | 0.0525 | 0.0862 |
| Bupivacaine | 0.10 | 0.0254 | 0.0590 |
| Ropivacaine | 0.20 | 0.0441 | 0.0805 |
| Negative control | 0 | 0.0087 | 0.0165 |

# Results from Quantum chemical calculations

**Table 4S** presents the results of quantum chemical calculations of radical reactions of lidocaine with the peroxyl radical in water. All results are calculated in kcal/mol and represent the free energy of the reaction. The bond position references possible reaction paths, indicated in **Fig. 1**.

**Table 4S: Free energy of reaction of lidocaine with peroxyl radical**

|  | **Mechanism** | | | | |
| --- | --- | --- | --- | --- | --- |
|  | HAT | SEP-PT | | SPLET | |
| **Bond position** | ΔG_HAT_ | ΔG_SEP-PT 1_ | ΔG_SEP-PT 2_ | ΔG_SPLET 1_ | ΔG_SPLET 2_ |
| 1 | 11.47 | 22.91 | -11.44 | 2.92 | 8.55 |
| 2 | -3.16 | 22.91 | -26.06 | 20.4 | -23.55 |
| 3 | 3.47 | 22.91 | -19.44 | 52.67 | -49.2 |
| 4 | 11.8 | 22.91 | -11.11 | 49.1 | -37.3 |
| 5 | 3.4 | 22.91 | -19.51 | 31.29 | -27.89 |
| 6 | 24.15 | 22.91 | 1.24 | 42.62 | -18.47 |
| 7 | 22.98 | 22.91 | 0.07 | 42.49 | -19.51 |

The values for the free energy of the reaction were calculated using the Minnesota functional of DFT at the level of theory M06-2X/6-311+G(d,p) and SMD solvation model. All results are calculated in kcal/mol and represent the reaction free energy. HAT – hydrogen atom transfer, SEP-PT - single electron transfer followed by proton transfer, SPLET - sequential proton loss electron transfer. Position 2 is the -C-H bond next to the carbonyl group (**Fig. 1**).

**Table 5S** presents the results of quantum chemical calculations of radical reactions of bupivacaine with the peroxyl radical in water. All results are calculated in kcal/mol and represent the free energy of the reaction.

**Table 5S: Free energy of reaction of bupivacaine with peroxyl radical**

|  | **Mechanism** | | | | |
| --- | --- | --- | --- | --- | --- |
|  | HAT | SEP-PT | | SPLET | |
| **Bond position** | ΔG_HAT_ | ΔG_SEP-PT 1_ | ΔG_SEP-PT 2_ | ΔG_SPLET 1_ | ΔG_SPLET 2_ |
| 1 | 13.6 | 37.86 | -24.26 | 3.18 | 10.42 |
| 2 | -0.24 | 37.86 | -38.1 | 29.72 | -29.96 |
| 3 | 10.09 | 37.86 | -27.77 | 50.7 | -40.61 |
| 4 | 10.68 | 37.86 | -27.18 | 53.73 | -43.05 |
| 5 | 10.76 | 37.86 | -27.1 | 52.41 | -41.65 |
| 6 | 5.16 | 37.86 | -32.7 | 54.75 | -49.59 |
| 7 | 5.62 | 37.86 | -32.24 | 55.05 | -49.43 |
| 8 | 8.25 | 37.86 | -29.61 | 54.39 | -46.14 |
| 9 | 9.23 | 37.86 | -28.63 | 56.58 | -47.35 |
| 10 | 11.84 | 37.86 | -26.02 | 54.23 | -42.38 |
| 11 | 4.36 | 37.86 | -33.5 | 32.71 | -28.35 |
| 12 | 23.84 | 37.86 | -14.02 | 43.82 | -19.97 |
| 13 | 23.81 | 37.86 | -14.05 | 43.41 | -19.6 |
| 14 | 23.84 | 37.86 | -14.02 | 43.82 | -19.97 |
| 15 | 4.48 | 37.86 | -33.38 | 32.51 | -28.02 |

The values for the free energy of the reaction were calculated using the Minnesota functional of DFT at the level of theory M06-2X/6-311+G(d,p) and SMD solvation model. All results are calculated in kcal/mol and represent the reaction free energy. HAT – hydrogen atom transfer, SEP-PT - single electron transfer followed by proton transfer, SPLET - sequential proton loss electron transfer. Position 2 is the -C-H bond next to the carbonyl group (**Fig. 1**).

**Table 6S** presents the results of quantum chemical calculations of radical reactions of ropivacaine with the peroxyl radical in water. All results are calculated in kcal/mol and represent the free energy of the reaction. The calculation at position 12 did not render even after several repetitions.

**Table 6S: Free energy of reaction of ropivacaine with peroxyl radical**

|  | **Mechanism** | | | |  |
| --- | --- | --- | --- | --- | --- |
|  | HAT | SEP-PT | | SPLET | |
| **Bond position** | ΔG_HAT_ | ΔG_SEP-PT 1_ | ΔG_SEP-PT 2_ | ΔG_SPLET 1_ | ΔG_SPLET 2_ |
| 1 | 12.01 | 37.4 | -25.4 | 1.88 | 10.13 |
| 2 | -1.71 | 37.4 | -39.11 | 28.55 | -30.26 |
| 3 | 9.71 | 37.4 | -27.69 | 50.11 | -40.4 |
| 4 | 9.95 | 37.4 | -27.46 | 53.18 | -43.24 |
| 5 | 10.17 | 37.4 | -27.24 | 51.36 | -41.19 |
| 6 | 4.06 | 37.4 | -33.34 | 53.96 | -49.89 |
| 7 | 5.39 | 37.4 | -32.01 | 53.19 | -47.8 |
| 8 | 7.3 | 37.4 | -30.11 | 54.23 | -46.93 |
| 9 | 11.34 | 37.4 | -26.06 | 52.11 | -40.77 |
| 10 | 3.38 | 37.4 | -34.02 | 31.57 | -28.19 |
| 11 | 23.64 | 37.4 | -13.77 | 43.06 | -19.42 |
| 12 | / | / | / | / | / |
| 13 | 23.45 | 37.4 | -13.95 | 42.89 | -19.44 |
| 14 | 3.37 | 37.4 | -34.04 | 31.57 | 28.2 |

The values for the free energy of the reaction were calculated using the Minnesota functional of DFT at the level of theory M06-2X/6-311+G(d,p) and SMD solvation model. All results are calculated in kcal/mol and represent the reaction free energy. HAT – hydrogen atom transfer, SEP-PT - single electron transfer followed by proton transfer, SPLET - sequential proton loss electron transfer. Position 2 is the -C-H bond next to the carbonyl group (**Fig. 1**).
